# Negative impact of malignant effusion on osimertinib treatment for non-small cell lung cancer harboring *EGFR* mutation

**DOI:** 10.1007/s10637-019-00808-1

**Published:** 2019-06-10

**Authors:** Takahisa Kawamura, Hirotsugu Kenmotsu, Haruki Kobayashi, Shota Omori, Kazuhisa Nakashima, Kazushige Wakuda, Akira Ono, Tateaki Naito, Haruyasu Murakami, Keita Mori, Masahiro Endo, Toshiaki Takahashi

**Affiliations:** 1 Division of Thoracic Oncology, Shizuoka Cancer Center, 1007 Shimonagakubo, Nagaizumi-cho, Suntou-gun, Shizuoka, 411-8777 Japan; 2 Clinical Research Center, Shizuoka Cancer Center, 1007 Shimonagakubo, Nagaizumi-cho, Suntou-gun, Shizuoka, 411-8777 Japan; 3 Division of Diagnostic Radiology, Shizuoka Cancer Center, 1007 Shimonagakubo, Nagaizumi-cho, Suntou-gun, Shizuoka, 411-8777 Japan

**Keywords:** Non-small cell lung cancer, EGFR-TKI, Osimertinib, Malignant effusion

## Abstract

3rd-generation epidermal growth factor receptor-tyrosine kinase inhibitors (EGFR-TKIs), including osimertinib, have reasonable efficacy in non–small-cell lung cancers (NSCLC) with *EGFR* mutations. However, the efficacy of osimertinib in NSCLC patients with fluids, such as pleural, pericardial and abdominal effusions, is unclear. We evaluated the efficacy of osimertinib in this specific setting. NSCLC patients harboring *EGFR* T790 M mutations who experienced progressive disease after first EGFR-TKI treatment and started osimertinib treatment between April 2016 and August 2018 were retrospectively screened. In particular, we assessed the efficacy of osimertinib for NSCLC with *EGFR* T790 M mutations in patients who were diagnosed with *EGFR* T790 M mutation by malignant effusion. Among 90 patients with *EGFR* T790 M mutation who started osimertinib treatment after EGFR-TKI failure, 21 were diagnosed from malignant effusions excluding cerebrospinal fluid (F group) and 69 using other methods including tissue biopsies (NF group). Patient characteristics were well-balanced between the two groups. Overall response was 50%, and significantly worse in the F group (29%) than the NF group (57%; *P* = 0.025). Median progression-free survival with osimertinib treatment in the F group (7.1 months, 95% confidence interval [CI]: 2.3–14.0) was significantly shorter than that in the NF group (11.9 months, 95% CI: 9.5–16.0; *P* = 0.046)). Median drainage-free time was 10.9 months (95% CI: 1.4 months– not reached). The present study showed that the efficacy of osimertinib for NSCLC in which *EGFR* T790 M mutation is detected by malignant effusion may be less than in *EGFR* T790 M-mutated NSCLC detected by other methods.

## Introduction

Non-small cell lung cancer (NSCLC) is a common cause of cancer-related death [1]. The identification of epidermal growth factor receptor (EGFR) as a driver oncogene has dramatically effected lung cancer treatment strategy. EGFR-tyrosine kinase inhibitors (TKIs; EGFR-TKIs) have produced potent responses in patients with *EGFR*-mutant NSCLC [2]. However, patients with advanced NSCLC that harbor *EGFR* mutations develop progressive disease (PD) after a median response period of 11 months [3]. A specific point mutation within exon 20 (T790 M) accounts for 30–60% of instances of acquired resistance to EGFR-TKI [4–8]. Osimertinib, a third-generation EGFR-TKI, is reportedly effective against NSCLC that harbors *EGFR* T790 M mutation, and was approved as a standard therapy after first EGFR-TKI failure [9–11]. However, limited information is available about its efficacy for EGFR-mutated NSCLC, especially in cases with associated body fluids, such as malignant pleural effusion, pericardial effusion, and ascites [12]. In this study, we focused on patients treated with osimertinib whose mutation status of *EGFR* T790 M was identified by fluid samples, including pericardial, abdominal and pleural effusion.

## Patients and methods

### Patients and *EGFR* mutation analysis

We retrospectively reviewed medical records of patients diagnosed with NSCLC that harbored *EGFR*-activating mutations and who received osimertinib treatment at Shizuoka Cancer Center from April 2016 to August 2018. For eligible patients, exon 20 T790 M mutation was detected by tissue samples and/or cytology samples and/or blood samples after the failure of at least one EGFR-TKI treatment. *EGFR-*activating mutations included exon 18 G719X mutation, exon 19 deletions, exon 20 S768I mutation, exon 21 L858R mutation, and exon 21 L861Q mutation. Patients with T790 M mutation detected in cerebrospinal fluid were excluded from our analyses because the emergence of T790 M in central nervous system is rare compared with other lesions, and leads to uncommon prognosis [13]. We assessed patient characteristics, efficacy of osimertinib including overall response rate (ORR), and progression-free survival (PFS). Efficacy data were compared between two subgroups: (a) patients in whom T790 M mutation was detected via malignant effusions vs other specimen types, including plasma samples; and (b) patients with vs without malignant effusion, based on radiographic evaluation. PFS was defined as the period from the date of initial osimertinib treatment to the date of PD. Duration of drainage-free time was defined as the date of initial osimertinib treatment to the day of next drainage time, because of symptoms such as dyspnea. For radiographic evaluation of malignant effusion, massive effusion detectable by chest radiograph (requiring drainage) or effusion of ≥10 mm thickness at computed tomography, were defined as third space fluid accumulation (patients with effusions), as previously reported [14]. We used Cobas *EGFR* Mutation Test kits version 2 in *EGFR* mutation analyses of tissue and cytology samples.

### Statistical analysis

Statistical analysis was performed using JMP 10 software (SAS Institute, Inc., Cary, NC, USA). Univariate analyses, using chi-squared and Mann–Whitney *U* tests, were used to evaluate differences in efficacy between the group in whom *EGFR* T790 M mutation was detected by fluid samples, and the group that used non-fluid samples. *P* < 0.05 was considered significant. This study was approved by the institutional review board of Shizuoka Cancer Center.

## Results

### Patient characteristics

We screened 92 patients with NSCLC who started osimertinib treatment after EGFR-TKI failure between April 2016 and August 2018. Among these patients, *EGFR* T790 M mutation was detected in 23 patients via body fluids (19 pleural effusion, 2 ascites, and 2 cerebrospinal fluid), and in 69 patients in other specimen types, such as primary lesions, lymph node metastases, other tissue samples and plasma samples. Two patients in whom T790 M mutation was detected by cerebrospinal fluid (other effusions were not identified radiographically in both the cases) were excluded from the analysis. Therefore, 21 T790 M-positive patients detected by fluid samples (F group) and 69 T790 M-positive patients detected by non-fluid samples (NF group) were analyzed in this study. Baseline patient characteristics (age at initiation of osimertinib treatment, gender, smoking status, performance status [PS], *EGFR* mutation type, surgical history, and number of previous chemotherapy regimens) are shown in Table 1. The Median age was 71 (range; 60–84) in F group and 68 (range; 38–89) in NF group, respectively. Patient characteristics were well-balanced between two groups. F group included two cases of uncommon mutations (compound mutation of ex18 G719X and ex20 S768I); the NF group did not. 5 patients in F group had massive effusion detectable just by chest radiograph, and 85 patients (16 in F group and 69 in NF group) were available for computed tomographic assessment immediately before osimertinib treatment. In the computed tomography, the effusion was identified in 16 F group patients and in 21 of 69 NF group patients. Median effusion thickness at computed tomography significantly differed between the F group (39 mm, range: 12–80) and the NF group (18 mm, range: 11–63; *P* = 0.0022). The previous history of pleurodesis was found in one case in the F group. Anatomical sites of progression after initial EGFR-TKI treatment are also shown in Table 1.The most common progressive lesion after initial EGFR-TKI treatment (gefitinib, erlotinib, and afatinib) in the F group was malignant effusion: 67% (*n* = 14), in contrast to 9% (*n* = 6) in NF group.

**Table 1:** Patient characteristics

	F group; *n* = 21	NF group; *n* = 69	*P*
Median age at start of osimertinib treatment (range)	71 (60–84)	68 (38–89)	0.28
Gender			0.94
Male	5	17	
Female	16	52	
Smoking status			0.67
Smoker	9	26	
Non-smoker	12	43	
PS at start of osimertinib treatment			0.78
0	2	13	
1	13	37	
2	5	15	
3	1	4	
*EGFR* mutation			
Exon 19 deletion	12	44	
Exon 21 L858R	7	25	
Others	2	0	
Surgical history			0.69
No; Advanced (III–IV)	17	53	
Yes; Post-surgery recurrence	4	16	
Median previous chemotherapy regimens (range)	3 (2–12)	3 (2–11)	0.98
Previous history of pleurodesis	1	0	
Anatomical progressive disease sites after initial EGFR-TKI treatments; *n* (%)			
Pleural effusion/ Ascites	14 (67)	6 (9)	
Thoracic lesion	9 (43)	50 (72)	
Bone lesion	3 (14)	16 (23)	
Brain lesion	5 (24)	16 (23)	
Liver lesion	0 (0)	11 (16)	
Others	14 (67)	29 (42)	
Malignant effusion in radiographic assessment			
Yes	21	21	
No	0	48	
Effusion thickness (mm, per computed tomography)	*n* = 16	*n* = 21	0.0022*
Median (range)	39 (12–80)	18 (11–63)	

*EGFR* epidermal growth factor receptor; F group: patients with NSCLC in which T790 M mutation was detected by fluid samples; *NF group* patients with NSCLC in which T790 M mutation was detected by other methods; *NSCLC* non-small-cell lung cancer; *PS* performance status

### Efficacy

**Fig. 1:**
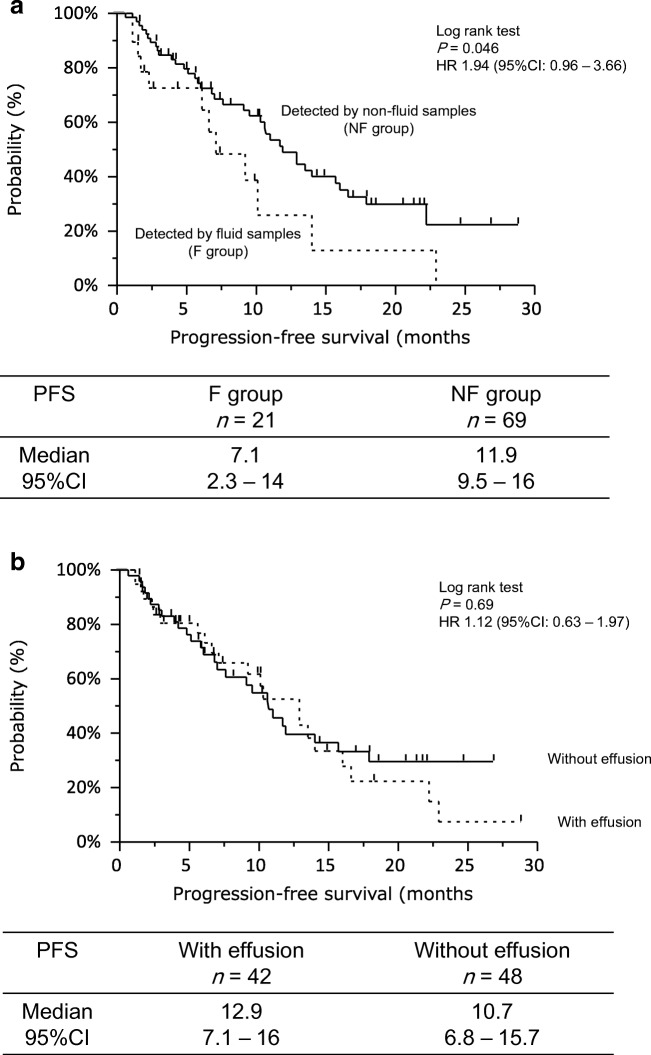
**a** Progression-free survival curves for osimertinib-treated patients with non-small-cell lung cancer that harbors T790 M mutation, which was detected in fluid samples (F group) or through other methods (NF group). **b** Comparison of progression-free survival curve of osimertinib-treated patients with or without effusions, based on radiographic evaluation

**Fig. 2:**
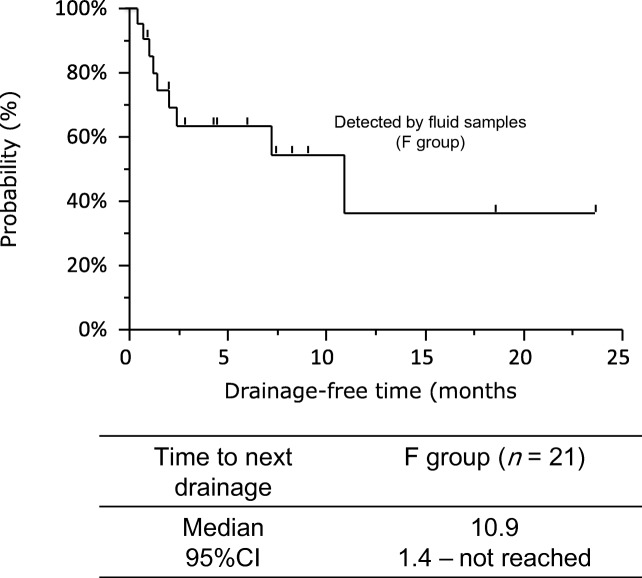
Drainage-free time curve of osimertinib-treated patients with non-small-cell lung cancer that harbors T790 M mutation, which was detected in fluid samples (F group)

Objective responses are shown in Table 2. Overall response was significantly worse in F group than in NF group (29% vs 57%, *P* = 0.025). Furthermore, median PFS with osimertinib treatment was significantly shorter in F group (7.1 months [95% confidence interval (CI): 2.3–14.0]) than in NF group (11.9 months [95% CI: 9.5–16.0]; *P* = 0.046; Fig. 1a). However, PFS did not significantly differ between patients with effusions (12.9 months [95% CI: 7.1–16.0]) and without effusions (10.7 months [95% CI: 6.8–15.7]; *P* = 0.69; Fig. 1b). In the F group, median drainage-free time was 10.9 months (95% CI: 1.4 months–not reached; Fig. 2).

**Table 2:** Overall responses

	F group; *n* = 21		NF group; *n* = 69		*P*
	*n*	%	*n*	%	
CR	0	0	0	0	
PR	6	29	39	57	
SD	6	29	20	29	
PD	5	23	5	7	
NE	4	19	5	7	
ORR		29		57	0.03*
DCR		58		86	0.02*
PD rate		23		7	0.02*

*CR* complete response; *DCR* disease control rate; *F group* patients with NSCLC in which T790 M mutation was detected by fluid samples; *NE* not evaluated; *NF group* patients with NSCLC in which T790 M mutation was detected by other methods; *ORR* overall response rate; *PD* disease progression; *PR* partial response; *SD* stable disease

### Progression pattern

Anatomical progressive disease sites after osimertinib treatment are shown in Table 3. In the F group, the most common progressive lesion following osimertinib treatment was malignant effusion at 43% (*n* = 9), in contrast to 10% (*n* = 7) in NF group.

**Table 3:** Anatomical progressive disease sites after osimertinib treatment

	F group; *n* = 21	NF group; *n* = 69
Anatomical progressive disease sites after osimertinib treatment	*n* (%)	*n* (%)
Pleural effusion/ Ascites	9 (43)	7 (10)
Thoracic lesion	5 (24)	20 (29)
Bone lesion	0 (0)	2 (3)
Brain lesion	3 (14)	8 (12)
Liver lesion	1 (5)	6 (9)
Others	4 (19)	14 (20)
Not evaluated	7 (33)	33 (48)

### Post-progression therapy

By the cutoff date, 9 (43%) of T790 M-positive patients in the F group and 33 (48%) in the NF group had not experienced disease progression after initiating osimertinib. Similarly, 6 patients (28%) in the F group and 13 patients (19%) in the NF group did not receive subsequent chemotherapy after osimertinib failure. Post-progression therapy after osimertinib failure is shown in Table 4. Among patients who could receive post-progression therapy, the most common regimen was platinum-doublet chemotherapy, for both groups.

**Table 4:** Post-progression therapies

	F group; *n* = 21		NF group; *n* = 69	
	*n*	%	*n*	%
Continuation of osimertinib	9	42	33	48
Cessation of osimertinib due to toxicity	1	5	2	3
EGFR-TKIs	0	0	1	1
Platinum doublet	2	10	8	12
Single non-platinum	1	5	4	6
Non-platinum + angiogenesis inhibitor	0	0	4	6
Immune checkpoint inhibitors	1	5	3	4
Investigational drugs	1	5	1	1
BSC	6	28	13	19

*BSC* best supportive care; *EGFR-TKI* epidermal growth factor receptor-tyrosine kinase inhibitor; *F group* patients with NSCLC in which T790 M mutation was detected by fluid samples; *NF group* patients with NSCLC in which T790 M mutation was detected by other methods

## Discussion

We conducted this retrospective study with the aim of evaluating osimertinib efficacy for patients with malignant effusions. Reportedly, osimertinib is efficacious for patients with *EGFR*-activating mutations, and is especially promising for those with central nervous system metastases. However, its efficacy toward fluid lesions such as malignant pleural and pericardial effusions, and ascites, has not been widely studied, though patients often suffer from symptoms such as dyspnea due to malignant effusions despite osimertinib treatment. A previous study reported that osimertinib monotherapy is less effective in patients with NSCLC with pleural effusions [12]. However, evidence is still scanty, as previous studies have been retrospective in design with small study cohorts. In the present study, median PFS did not significantly differ between patients with and without effusions: more appropriate evaluation of effusions (including the presence of malignancy and/or thickness in computed tomography) is needed in the analysis, although it is feasible only through prospective studies. The present study revealed that the median PFS in osimertinib-treated patients whose T790 M-positive status was detected by fluid samples was significantly shorter than if detected by non-fluid samples. Similarly, overall response in the F group was significantly worse than in NF group. One possible explanation involves osimertinib penetration to third space. As for other EGFR-TKIs, Masago et al. investigated the pharmacokinetics of erlotinib in NSCLC with malignant pleural effusion, and found repeated erlotinib dosing led to significant accumulating drug concentrations in malignant effusions, with the mean percentage of erlotinib penetration from plasma to pleural effusion to be 18% on Day 1 and 112% on Day 8 [15]. However, penetration from plasma to pleural effusion for osimertinib may be low, unlike erlotinib. Therefore, greater understanding of the pharmacokinetics or pharmacodynamics of osimertinib is critical. We are therefore conducting a prospective study that evaluates the association of malignant effusion with osimertinib exposure in NSCLC (UMIN000028922).

Another explanation concerns effusion quantity. In the present study, median effusion thicknesses at computed tomography in F group were greater than in NF group, and median PFS with osimertinib treatment in F group was significantly shorter than in NF group. On the other hand, PFS did not differ between patients with and without effusions by radiographic assessment. It suggests that negative impact of osimertinib efficacy may be due to the quantity of malignant effusions, not due to the radiographic presence of malignant effusions. However, cutoff values of the quantity remain unclear.

Our study showed that median drainage-free time was 10.9 months. This suggests that osimertinib has a certain efficacy in the management of effusion despite poor response rate in patients with malignant effusion.

This study has some limitations. First, despite the inclusion of larger-scale data than previous studies, it is limited by its retrospective design. Second, data were obtained from patients at a single institution. Finally, not all effusions detected radiologically in both F group and NF group were confirmed as T790 M positive in this study.

In conclusion, efficacy for osimertinib treatment in patients with *EGFR* T790 M mutations that were detected via malignant effusion may be limited compared with previous reports. An analysis with a larger cohort is needed to validate the results in this study.

## Abbreviations

CI: Confidence interval
EGFR: Epidermal growth factor receptor gene
NSCLC: Non-small-cell lung cancer
ORR: overall response rate
PD: progressive disease
PFS: Progression-free survival
PS: Performance status
TKI: Tyrosine kinase inhibitor
